# Cardioprotective and antioxidant effects of Rosa damascena leaf and stem extract against lead acetate-induced myocardial injury in rats

**DOI:** 10.3389/fphar.2026.1912345

**Published:** 2026-08-07

**Authors:** Sahar Jameel Melebary

**Affiliations:** Department of Biological Sciences, College of Science, University of Jeddah, Jeddah, Saudi Arabia

**Keywords:** cardioprotection, histopathology, lead acetate, oxidative stress, rats, Rosa damascena

## Abstract

Cardiovascular disease and environmental pollution are major global health concerns, with lead exposure posing a serious public risk through oxidative stress, fibrosis, and cardiac damage. This study was designed to evaluate the antioxidant and cardioprotective effects of Taif Rose (leaves and stems) extract against lead acetate–induced myocardial injury in rats. Forty adult male Wistar rats were divided into four groups (n = 10): control, Taif Rose extract (0.5 mg/kg/day), lead acetate (0.2 mg/kg/day), and lead acetate plus Taif Rose extract, treated orally for 30 days. Cardiac injury was quantitatively evaluated by measuring serum creatine kinase-MB (CK-MB) and cardiac troponin I (cTnI), together with cardiac oxidative stress biomarkers (MDA, GSH, and SOD), followed by histopathological and quantitative morphometric analyses. Lead exposure induced marked cardiotoxicity characterized by elevated CK-MB, cTnI, and MDA levels, decreased GSH and SOD activities, and significant histological alterations, including myocardial degeneration, inflammatory infiltration, and collagen deposition. Co-administration of Taif Rose extract significantly mitigated these effects, restoring antioxidant balance, reducing serum cardiac injury markers, and preserving normal myocardial structure with minimal fibrosis. The findings suggest that Rosa damascena leaf and stem extract may have potential therapeutic value against lead-induced cardiotoxicity in rats.

## 1 Introduction

Cardiovascular diseases (CVDs) represent the foremost cause of morbidity and mortality worldwide. According to the World Health Organization (WHO), cardiovascular diseases are responsible for an estimated 17.9 million deaths annually worldwide. The World Health Organization (WHO) projects that 80% of global annual fatalities will result from noncommunicable diseases by 2030 (Kumar et al., 2025). Environmental pollution and exposure to heavy metals constitute significant, although sometimes overlooked, contributors to cardiovascular disorders, particularly in comparison to other ailments within the general populace. The examination of the long-term health effects of environmental contamination through a multi-exposure framework known as the exposome idea (Lamas et al., 2023).

Lead (Pb) is a dense metal of historical significance. This metal is deemed an environmental contaminant due to elevated amounts of the element present in the atmosphere, soil, water, and food. Natural sources of lead include soil erosion and volcanic eruptions, nevertheless, anthropogenic sources are the predominant contributors (Gioda et al., 2022). The effects of lead exposure on cells, tissues, organs, and systems have been recorded. Anemia, a condition significantly associated with lead exposure, serves as a prominent example. Research has examined the impact of Pb on the lungs, kidneys, liver, cardiovascular system, reproductive system, and genotoxicity, demonstrating an elevated risk of genotoxicity and cancer (Abdulhai et al., 2024; Jomova et al., 2025).

Adverse effects persist at lower concentrations, especially in highly susceptible populations like children, who may experience attention deficits and cognitive, behavioral, and memory difficulties (Swaringen et al., 2022). The WHO has released a list of ten substances or chemical groups of concern for human health, which includes lead (Pb). The WHO has asserted that no level of exposure is deemed safe (World Health Organization W, 2024). The US Agency for Toxic Substances and Disease Registry (ATSDR) has rated lead (Pb) second on its priority list of hazardous compounds (Desai et al., 2024). Lead can induce oxidative stress, and growing evidence suggests that oxidative stress affects the pathophysiology of lead poisoning, known as plumbism. The immune system is consistently subjected to diverse environmental stressors and xenobiotics, including heavy metals such as lead, and is considered one of the most sensitive biological systems. Following lead exposure, cells experience oxidative stress due to the generation of reactive oxygen species (ROS). When the production and utilization of ROS are imbalanced, numerous cellular structures, especially phospholipids, are compromised, resulting in lipid peroxidation (Harshitha et al., 2024).

The left ventricle (LV), as the primary pumping chamber of the heart, is especially susceptible to toxic damage. Injury to the left ventricular myocardium not only diminishes cardiac output but also initiates compensatory remodeling processes, such as fibrosis and inflammatory infiltration. Many years after the initial studies suggested an association between lead (Pb) exposure and cardiovascular diseases, the mechanisms and its contribution to cardiovascular outcomes require further investigation (Chen et al., 2021), particularly in low-level environmental exposure scenarios (Protsenko et al., 2020). Thus, incorporating a moderate, sublethal dosage exposure is essential for investigating the cardiotoxic effects of Pb under realistic, environmentally relevant exposure conditions (Neha et al., 2025).

Recently, focus has turned to natural medicinal compounds possessing antioxidant and anti-inflammatory effects as potential safeguards against organ toxicity generated by heavy metals (Hakimi et al., 2025). Medicinal plants have attracted attention for their bioactive components, such as phenolic acids, flavonoids, and essential oils, which possess the ability to scavenge free radicals, augment endogenous antioxidant systems, and regulate inflammatory pathways (Riaz et al., 2023). *Rosa damascena* is a rich source of bioactive phytochemicals, including flavonoids (e.g., quercetin, kaempferol), essential oils (e.g., geraniol, citronellol), and phenolic compounds, which exhibit significant antiviral, antioxidant, anti-inflammatory, and antimicrobial properties (He and Putra, 2025).

Taif Rose has been studied for its general pharmacological effects, including hepatoprotective (Hamza et al., 2022), neuroprotective, and anti-inflammatory actions, its cardioprotective role, particularly in mitigating lead-induced myocardial injury, remains largely unexplored. Examining its impact on left ventricular histology and biochemical indicators of oxidative stress may yield significant insights into its therapeutic efficacy and underlying mechanisms. Comprehending these effects may aid in the creation of innovative, plant-derived solutions for heavy metal–induced cardiotoxicity, particularly pertinent in areas with elevated environmental lead exposure (ip).

Therefore, the present study was designed to evaluate the antioxidant and cardioprotective effects of Taif Rose (leaves and stems) extract against lead acetate–induced myocardial injury in rats.

## 2 Materials and methods

### 2.1 Preparation and administration of lead acetate solution

A fresh lead acetate solution (Sigma-Aldrich, Germany) was prepared daily by dissolving 2 mg in 10 mL of distilled water to obtain a concentration of 0.2 mg/mL. Rats received 1 mL/kg body weight via oral gavage, corresponding to a final dose of 0.2 mg/kg/day. This dose was selected to represent a low, sublethal exposure level consistent with previous experimental studies. Supporting literature indicates that similar or lower doses have been used to induce measurable biological effects without causing acute mortality (Al-Juboori et al., 2016; Gosselin et al., 1984).

### 2.2 Preparation and administration of Taif Rose extract

Fresh leaves and stems of Rosa damascena (Taif Rose) were harvested from a rose cultivation in Taif City, located in Saudi Arabia’s Al-Hada Highland. The botanical materials were shade-dried at ambient temperature and pulverized into a fine powder utilizing an electric grinder. The powdered substance was extracted with 70% ethanol utilizing a Soxhlet apparatus. The extract was further filtered and concentrated under reduced pressure at 40 °C utilizing a rotary evaporator to yield a semisolid residue, which was preserved at −20 °C in airtight containers until required (Hamza et al., 2022). The crude ethanolic extract was prepared using a standardized extraction protocol and was used consistently throughout all experimental procedures.

The requisite dosage for delivery was freshly made each day by dissolving the extract in 0.9% normal saline and administered orally via gavage at a dosage of 0.5 mg/kg body weight each day. This dosage is within the established safety range reported in previous studies, representing merely 0.025%–0.05% of the documented oral LD_50_ values in rats, which span from 2 to 6 g/kg (Lalovski et al., 2025). A low, sublethal dose guarantees animal safety during prolonged treatment while facilitating the assessment of potential cardioprotective and biochemical effects.

## 3 Animals

The current investigation was conducted on healthy adult male Wistar albino rats (n = 40, aged approximately 20 weeks, weighing 180–250 g). Male rats were selected to minimize hormonal variability associated with the female estrous cycle and ensure experimental consistenc. The rats were acquired from the Animal House of the King Fahd Center for Medical Research at King Abdulaziz University, Jeddah, which served as the venue for all animal studies and sample collection. The sample size (n = 10 per group) was selected based on previous published experimental studies using similar animal models of lead-induced cardiotoxicity and antioxidant intervention. Throughout the study duration, the animals were housed in designated stainless-steel cages (three rats per cage) and maintained in an air-conditioned environment under standard laboratory conditions [alternating 12-h light and dark cycles, relative humidity of 50%–60%, and a temperature of 23 °C ± 2 °C]. The rats were provided with standard rodent food pellets and water *ad libitum*. Before the commencement of any experimentation, a period of 7 days for acclimatization was allowed. The animal experiments were conducted at King Fahd Medical Research Center, King Abdulaziz University, Jeddah, Saudi Arabia. The experimental protocol was reviewed and approved by the Institutional Animal Care and Use Committee (IACUC) of Zagazig University, Egypt (Approval No. ZU-IACU/3/F/518/2025), and all procedures were performed in accordance with the approved guidelines.

### 3.1 Experimental design

The animals were randomly assigned to four groups (n = 10 each) and treated for 30 consecutive days as follows.

Group I (Control): Rats received oral normal saline (0.9% NaCl) via gavage.

Group II (Taif Rose extract): Rats received Taif Rose extract (0.5 mg/kg body weight/day) orally via gavage, a dose selected based on prior reports demonstrating antioxidant efficacy and safety in experimental models. Additionally, a preliminary pilot study confirmed that this dose was effective in producing biological and histological effects (Al-Juboori et al., 2016).

Group III (Lead acetate): Rats received lead acetate (0.2 mg/kg body weight/day) orally via gavage.

Group IV (Lead acetate + Taif Rose extract): Rats received lead acetate (0.2 mg/kg/day, orally) first, followed 15–30 min later by Taif Rose extract (0.5 mg/kg/day, orally) via gavage. This schedule was repeated once daily for 30 consecutive days to allow the extract to exert potential protective effects against lead-induced cardiotoxicity and oxidative stress.

### 3.2 Blood and tissue collection

Following the experimental phase, the rats underwent an overnight fast. The rats were weighed 24 h post their final drug administration. Subsequently, rats were anesthetized with an intraperitoneal injection of thiopental at a dosage of 50 mg/kg body weight to establish general anesthesia at the conclusion of each research session.

Venous blood samples (3 mL per rat in each study group) were collected from the retro-orbital plexus utilizing capillary glass tubes. The collected blood was allowed to coagulate at ambient temperatures for 30 min. The serum was subsequently isolated via centrifugation for a minimum of 15 min at 3,000 g and 4 °C. The separated serum was stored at −80 °C until utilized (Etten, 2002).

The animals were subsequently euthanized through decapitation. Rats in each group underwent.

The thoracic cavity was incised, the heart was promptly removed, and it was rinsed in an ice-cold saline solution. Their hearts were excised, blotted dry, and weighed using an electronic balance. Subsequently, the left ventricle was longitudinally dissected and divided into two sections, one section was further cut into 5 mm^3^ fragments, preserved in 10% neutral-buffered formalin (pH = 7.4) for 24 h, and then processed conventionally to produce paraffin blocks. The blocks were made by washing in 0.1 M phosphate-buffered saline, dehydrating in increasing concentrations of alcohol, clearing, and subsequently embedding in paraffin. Conversely, the remaining left ventricular tissue slice was utilized for a tissue homogenate for biochemical testing. All biochemical assays were performed according to the manufacturers’ instructions using standardized laboratory procedures. Histopathological evaluation was conducted independently by two blinded histopathologists. All biochemical assays were performed in triplicate to ensure analytical reliability.

### 3.3 Serum CK-MB and troponin I (cTnI) assay estimation

Serum creatine kinase–myocardial band (CK-MB) activity and cardiac troponin I (cTnI) are well-established biomarkers of myocardial injury (Radhiga et al., 2012). Serum CK-MB activity and cTnI concentrations were determined using commercially available assay kits (Boster Biological Technology, Wuhan, China) according to the manufacturer’s instructions.

### 3.4 Light microscopic examination of the left ventricle

Paraffin sections of 5 µm thickness were cut using a rotary microtome and mounted on glass slides, and were handled for histological analysis under a light microscope (Suvarna et al., 2018). Sections were stained with hematoxylin and eosin (H&E) for a comprehensive histological evaluation of cardiac structure, cellular integrity, and indications of necrosis or inflammation. Hematoxylin has a deep blue-purple color and stains nucleic acids. Eosin is pink and stains proteins nonspecifically. In a typical tissue, nuclei are stained blue, whereas the cytoplasm and extracellular matrix have varying degrees of pink staining. Sections were stained using Masson’s trichrome stain to assess collagen fiber deposition and cardiac fibrosis. Histological slides were analyzed using a light microscope (Olympus BX53, Japan) by two blinded histopathologists, and representative images were obtained using hematoxylin and eosin.

### 3.5 Preparation of left ventricular tissue homogenates and oxidative stress parameters evaluation

The left ventricular tissue was homogenized (10% w/v) in ice-cold phosphate buffer (0.1 M, pH 7.4) and subsequently centrifuged at 10,000 rpm for 15 min at 4 °C. The protein concentration in the supernatant was quantified using the Lowry technique (Lowry et al., 1951) and calibrated for subsequent tests. The supernatants from tissue homogenates were utilized to quantify myocardial concentrations of malondialdehyde (MDA), a by-product of lipid peroxidation, according to the method of Janicka et al. (2024), and reduced glutathione (GSH) levels using the method of Tietz (Tietz, 1969). Furthermore, superoxide dismutase (SOD) activity was evaluated based on the method of Yilgor and Demir (2024). The Oxidative Stress Parameters assay kits from Boster Biological Technology, Wuhan, China, were employed according to the manufacturer’s instructions.

### 3.6 Histomorphometry analysis

Histological images were acquired using a Leica microscope equipped with the Leica Qwin 500 image analysis system (Leica Microsystems Imaging Solutions Ltd., Cambridge, UK). Quantitative morphometric analysis of the collagen area percentage in Masson’s trichrome-stained sections was subsequently performed using ImageJ software (NIH, Bethesda, MD, USA). Ten randomly selected, non-overlapping fields per section were analyzed at ×400 magnification, and the mean value ± standard deviation (SD) was calculated for each specimen (Janicka et al., 2024).

### 3.7 Statistical analysis

Data were analyzed using GraphPad Prism 10.0 (GraphPad Software, USA). Results were expressed as mean ± standard deviation (SD). Normality was assessed using the Shapiro-Wilk test. Comparisons between groups were performed using one-way analysis of variance (ANOVA), followed by Tukey’s *post hoc* test. A p-value < 0.05 was considered statistically significant.

## 4 Results

### 4.1 General observations

All rats completed the study without mortality until the end of the experimental period. Rats subjected to lead acetate demonstrated symptoms of overall weakness, reduced activity, and a dull hair coat compared to the control group. Histological examination of myocardial sections from both Group I (control) and Group II (Taif Rose extract) revealed no discernible structural alterations, and the normal architecture of the cardiac muscle fibers was preserved. Both groups had comparable ranges, with no significant differences in any of the examined morphometric characteristics (P > 0.05). Group I was chosen to give the results of the control group, which was employed in the statistical analysis, compared to other groups.

**FIGURE 1:**
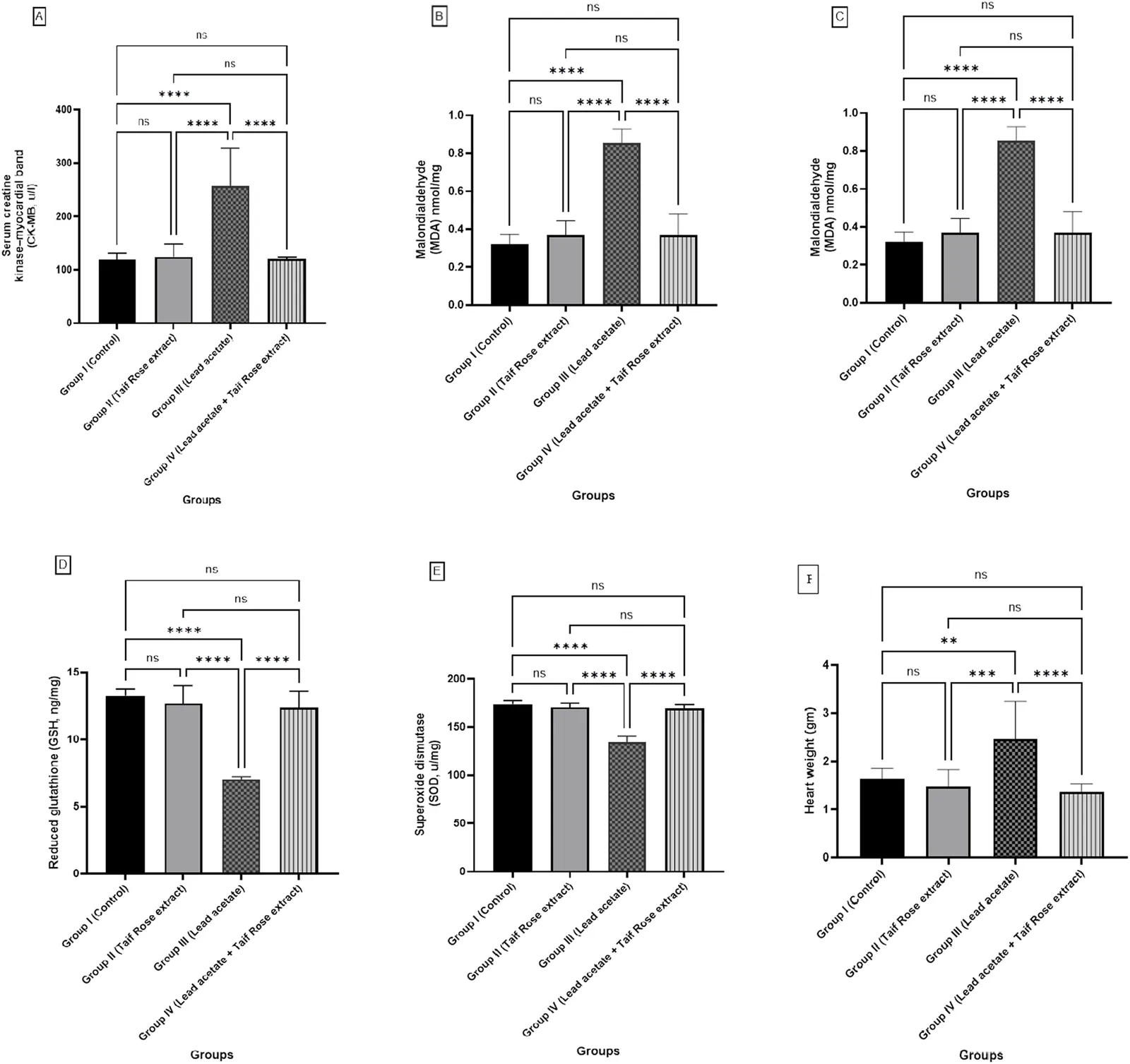
Effect of Rosa damascena (Taif Rose) extract on serum cardiac injury biomarkers: **(A)** creatine kinase–myocardial band (CK-MB) and **(B)** cardiac troponin I (cTnI), and on cardiac tissue oxidative stress biomarkers: **(C)** malondialdehyde (MDA), **(D)** reduced glutathione (GSH), **(E)** superoxide dismutase (SOD), and **(F)** heart weight. Data are presented as mean ± standard deviation (SD). Statistical analysis was performed using one-way ANOVA followed by Tukey’s multiple comparisons test. Significance levels are indicated as follows: ns, not significant; P < 0.05; P < 0.01; P < 0.001; P < 0.0001. Exact adjusted P values are reported in the Results section.

The lead acetate-treated group (Group III) exhibited a significant increase in heart weight (2.467 ± 0.7804 g) compared with both the control group (1.648 ± 0.2063 g; P = 0.0013) and the Taif Rose extract group (1.475 ± 0.3520 g; P = 0.0001). In contrast, co-administration of Rosa damascena leaf and stem extract (Group IV) significantly reduced heart weight (1.363 ± 0.1696 g) compared with the lead acetate-treated group (P < 0.0001). Notably, heart weight in Group IV did not differ significantly from the control group (P = 0.4947), indicating that Taif Rose extract effectively attenuated lead-induced cardiac enlargement and restored heart weight toward normal levels (Figure 1F).

### 4.2 Serum cardiac injury biomarkers (CK-MB and troponin I (cTnI))

Figures 1A,B demonstrate that lead acetate exposure resulted in a significant (P < 0.0001) increase in serum CK-MB (256.3 ± 71.62) and troponin I (cTnI) (48.15 ± 3.492) levels compared to the control group (118.9 ± 12.92 and 24.65 ± 5.082, respectively), signifying marked myocardial cell membrane damage and the release of cardiac enzymes into the bloodstream. Treatment with Taif Rose extract alone (Group II) did not induce any significant changes (P = 0.9920 and P = 0.1428, respectively) in serum CK-MB and troponin I (cTnI) levels (123.8 ± 25.23 and 29.24 ± 5.734, respectively) compared with the control group, indicating its safety under the tested conditions.

Co-administration of Taif Rose extract with lead acetate significantly decreased CK-MB and troponin I (cTnI) levels (120.8 ± 2.656 and 28.96 ± 4.028, respectively) compared to the lead-only group (P < 0.0001), indicating a significant protective effect on cardiac parameters.

### 4.3 Oxidative stress biomarkers in cardiac tissue

Exposure to lead acetate (Group III) resulted in a substantial (P < 0.0001) elevation in myocardial MDA levels (0.8540 ± 0.07367) relative to the control group (0.3220 ± 0.05138), signifying increased lipid peroxidation (Figure 1C). A significant reduction in glutathione (GSH) and superoxide dismutase (SOD) activity was also observed (Figures 1D, E) to the control group (13.26 ± 0.5254 and 174.0 ± 3.559, respectively), indicating depletion of endogenous antioxidant defences. In contrast, rats administered Taif Rose extract in conjunction with lead acetate (Group IV) exhibited a significant decrease in MDA levels (0.3700 ± 0.1106) and a restoration of GSH (12.36 ± 1.215) and SOD activity (168.9 ± 4.818) relative to the lead-only group (Group III). The results validate the antioxidant efficacy of Taif Rose extract in alleviating lead-induced oxidative stress in cardiac tissue. Treatment with Taif Rose extract alone (Group II) did not induce any significant alterations (P = 0.5475, P = 0.5516, and P = 0.4024, respectively) in MDA, GSH, and SOD biomarkers (0.3700 ± 0.07468, 12.70 ± 1.327, and 170.6 ± 4.377, respectively) when compared to the control.

#### 4.3.1 Histopathological findings

##### 4.3.1.1 Histological examination of H&E-stained cardiac sections

**FIGURE 2:**
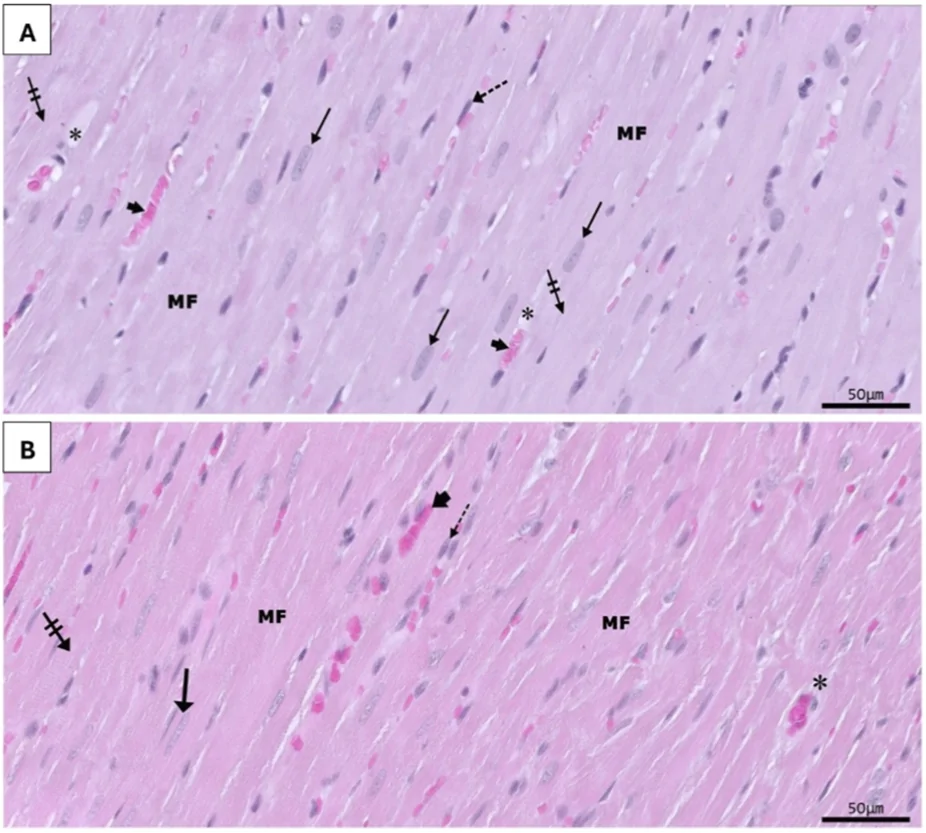
Representative photomicrographs of longitudinal sections of left ventricular myocardium of the control group I **(A)** and Taif Rose extract group II **(B)**, demonstrating branching and anastomosing cardiac muscle fibers with myofibrils (MF) parallel to the muscle fibers’ longitudinal axis. It exhibits acidophilic sarcoplasm with transverse striations (double-struck arrow), centrally placed ovoid vesicular nuclei (black arrow), and thin interstitial CT spaces (*) containing blood capillaries (arrowhead) and fibroblasts with flat dark nuclei (dot arrow). (H&E × 40).

Analysis of group I (Control) and group II (Taif Rose) revealed normal cardiac muscle architecture (Figures 2A,B, respectively). Light microscopy of the longitudinally sliced myocardium from control group I revealed the characteristic histological structure when cardiac tissues were stained with hematoxylin and eosin. The myocardium consists of regularly arranged cardiac fibers formed by myofibrils aligned parallel to the longitudinal axis of the muscle fibers. The cardiomyocytes possess centrally located oval vesicular nuclei and have a cylindrical morphology with acidophilic sarcoplasm. Furthermore, fibroblasts, characterized by darkly stained flattened nuclei, and blood capillaries were identified within the connective tissue interstices between cardiac muscle fibers.

**FIGURE 3:**
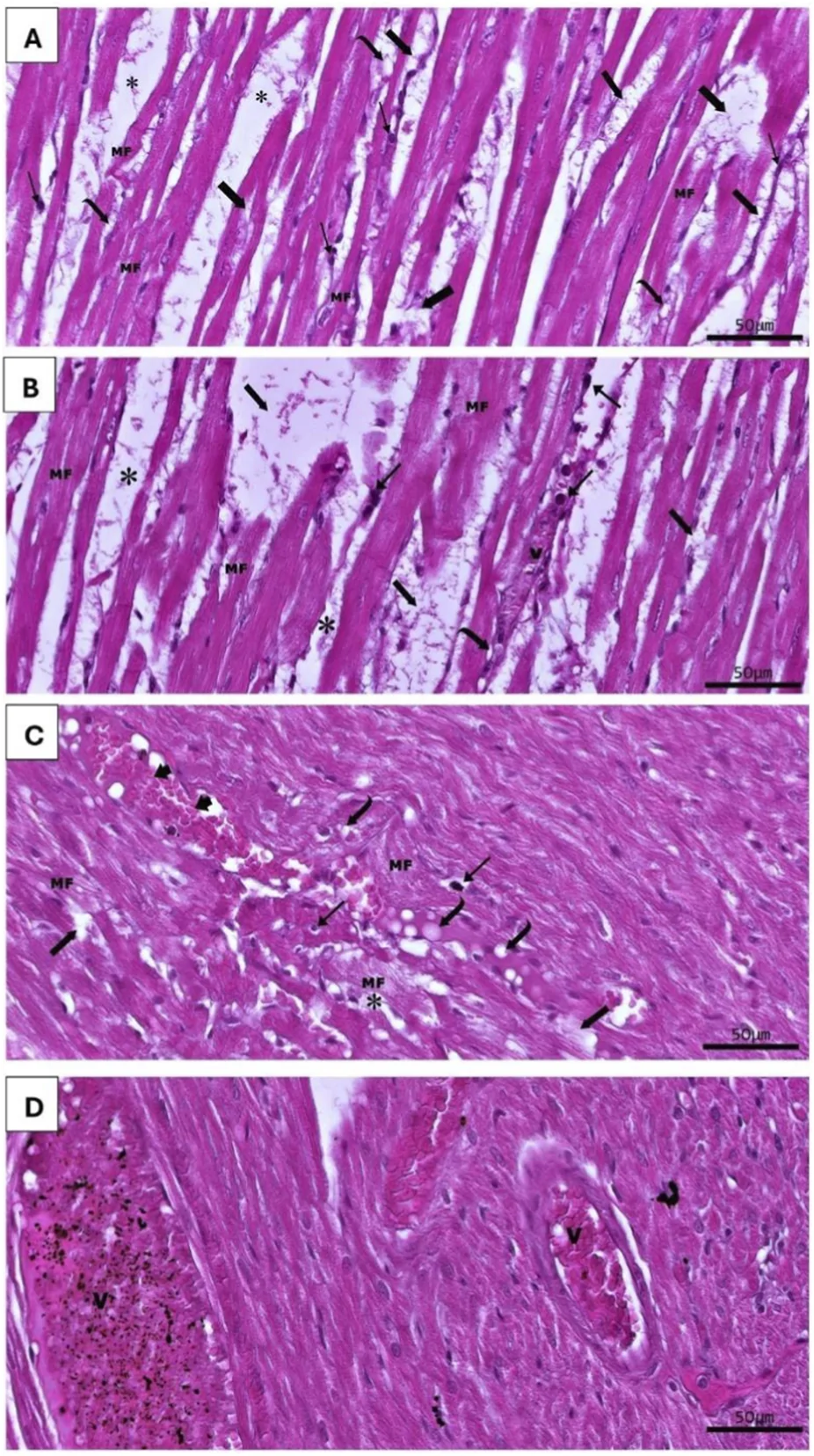
Representative photomicrographs of longitudinal sections of left ventricular myocardium of the lead acetate (Group III) demonstrating **(A,B)** loss of normal cardiac muscle fiber (MF) architecture with wide areas of complete loss of cardiac fibers (thick arrow) with darkly stained pyknotic nuclei of cardiomyocytes (thin arrow). Notice the wide separation (*) of cardiac muscle fibers. **(C)** Some cardiac muscle fibers (MF) appear shrunken and exhibit deeply stained homogenous acidophilic sarcoplasm and pyknotic nuclei (thin arrow), while others present perinuclear sarcoplasmic vacuolation (curved arrow). Notice fragmented, degraded muscle fibers (MF) with dilated, congested capillaries in-between (arrowhead) within the widened endomysium. **(D)** A congested blood vessel is also visible (V). (H&E × 40).

Compared to the control group, sections from rats treated with lead acetate (Group III) exhibited notable degenerative abnormalities characterized by discontinuity, extensive separation, and disorganization of heart muscle fibers. The endomysium was extensive between the heart muscle fibers. Certain cardiomyocytes appeared atrophied, displaying intensely pigmented homogeneous acidophilic sarcoplasm and pyknotic nuclei, whereas others exhibited perinuclear sarcoplasmic vacuolation. Additionally, dilated and congested capillaries within the expanded endomysium were clearly illustrated, with certain arteries exhibiting significant congestion (Figures 3A–D).

**FIGURE 4:**
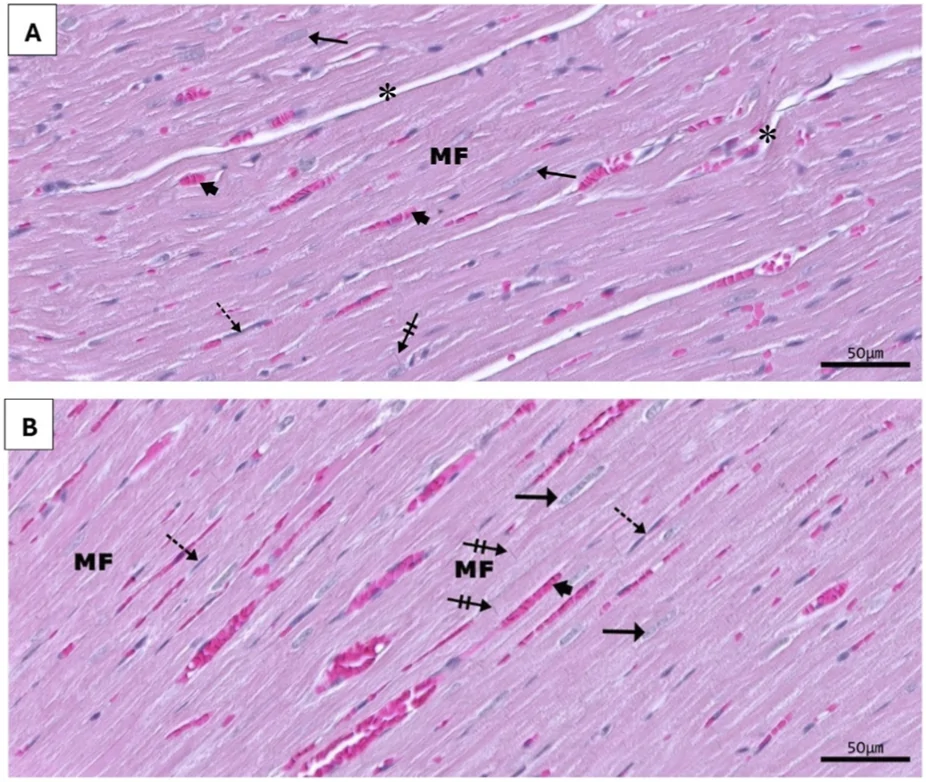
Representative photomicrographs of longitudinal sections of left ventricular myocardium of the lead + Taif Rose co-treated group (Group IV) demonstrating **(A,B)** remarkably preserved branching and anastomosing cardiac muscle fibers with myofibrils (MF) parallel to the muscle fibers’ longitudinal axis. It exhibits acidophilic sarcoplasm with transverse striations (double-struck arrow), centrally placed ovoid vesicular nuclei (black arrow), and thin interstitial CT spaces (*). A mildly congested blood vessel (arrowhead) between the muscle fibers is seen compared to the lead-only group. (H&E × 40).

The group treated with lead and Taif Rose exhibited significantly maintained cardiac fibers, a regular shape, and oval fibroblast nuclei interspersed throughout. Myocytes had centrally located oval nuclei. A somewhat obstructed blood vessel among the muscle fibers was observed in comparison to the lead-only group (Figures 4A,B). The findings suggest protective effect of Taif Rose extract against lead-induced cardiac degeneration.

##### 4.3.1.2 Collagen deposition and fibrosis (Masson’s trichrome staining)

**FIGURE 5:**
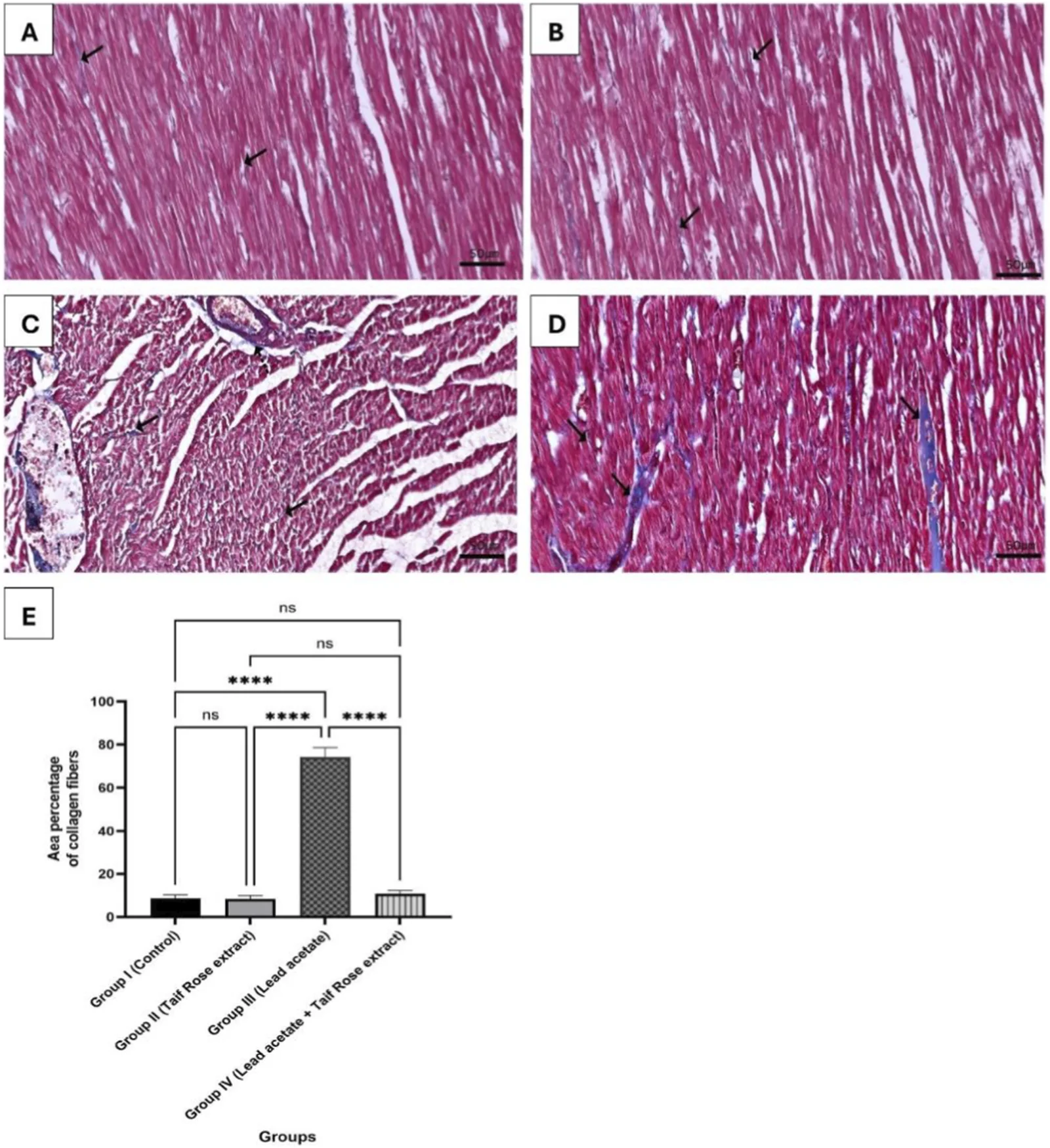
Representative photomicrographs of longitudinal sections of left ventricular myocardium stained with Masson’s Trichrome staining, demonstrating blue-colored collagen fibers in the connective tissue spaces between cardiomyocytes (↑) and around the blood vessels (dotted arrow) **(A,B)**: demonstrates delicate collagen fibers in the control group I **(A)** and Taif Rose extract group II **(B,C)** exhibits an increased amount of collagen fibers in the lead acetate (Group III). **(D)** Displays a lesser amount of collagen fibers in the recovery group. (Masson’s Trichrome stain × 20). **(E)** Bar charts demonstrating the quantitative analysis of collagen fiber area % in the experimental groups. Data are presented as mean ± SD. ns: non-significant and **** P < 0.0001.

Minimal collagen fibers were discernible between the cardiac muscle fibers in the endomysium of the control group when analyzed using light microscopy of Masson’s trichrome-stained slices (Figure 5A). The group treated with Taif Rose demonstrated collagen distribution similar to that of the control (Figure 5B). Masson’s trichrome-stained sections demonstrated a significant elevation in collagen fiber accumulation inside the interstitial and perivascular regions of the myocardium in the lead acetate group relative to the control (Figure 5C). A minimal quantity of collagen fiber production was observed among the fibers of the heart muscle in the group receiving co-administration with Taif Rose extract and lead acetate (Figures 4A, B).

Quantitative morphometric analysis utilizing the Leica Qwin 500 image analysis system and ImageJ software demonstrated a substantial (P < 0.0001) elevation in the mean area % of collagen fibers in lead acetate–exposed rats (74.18 ± 4.42) relative to the control group (8.62 ± 1.658). Conversely, rats administered Taif Rose extract (10.8 ± 1.398) exhibited significant reductions (P < 0.0001) in the percentage of collagen fiber area compared to the lead-only group, nearing normal levels. Treatment with Taif Rose extract alone (Group II) did not result in any significant alteration (P = 0.9949) in the percentage of collagen fiber area (8.34 ± 1.602) compared to the control, thus indicating its safety (Figure 5E).

## 5 Discussion

Chronic exposure to lead acetate induced marked cardiotoxic effects in male albino rats, as evidenced by structural alterations in the left ventricle, increased heart weight, elevated cardiac biomarkers (cTnI and CK-MB), enhanced lipid peroxidation, and depletion of antioxidant defenses. These findings suggest the development of oxidative stress-may mediated cardiac injury and compensatory myocardial hypertrophy. Similar observations were reported by Protsenko et al. (2018), who demonstrated that subchronic lead exposure increases myocardial mass through enhanced vascular resistance, disturbed calcium homeostasis, and oxidative stress. Lead toxicity is also known to disrupt calcium-dependent signaling pathways regulating myocardial contraction and relaxation, ultimately promoting hypertrophic remodeling of cardiac tissue. Previous studies have shown that acute lead exposure impairs cardiac function through alterations in Cav1.2 calcium channels (Ferreira de Mattos et al., 2017). Consistent with these observations, co-administration of Rosa damascena leaf and stem extract significantly ameliorated lead-induced.

In the present investigation, rats administered Taif Rose extract alongside lead acetate (Group IV) exhibited a notable decrease in heart weight relative to the lead-only group. This aligns with previous results indicating that herbal antioxidants like Rosa damascena and green tea polyphenols can inhibit or reverse toxin-induced cardiac hypertrophy by scavenging reactive oxygen species and regulating intracellular signaling (Hedayati et al., 2023). This discovery indicates that Taif Rose extract mitigated lead-induced cardiac hypertrophy, likely via its antioxidant, anti-inflammatory, and anti-fibrotic properties. Rosa damascena is recognized for its abundance of bioactive components, including flavonoids, phenolic acids, terpenes, and anthocyanins, which demonstrate free radical scavenging and cytoprotective properties (Riaz et al., 2023). The histological assessment of the left ventricular myocardium in this study demonstrated significant changes due to lead acetate exposure and a pronounced protective effect from the concurrent administration of Taif Rose (Rosa damascena) extract. In the control and Taif Rose-only groups, the myocardium displayed a typical histological pattern characterized by well-organized, parallel cardiac fibers, centrally positioned vesicular nuclei, and preserved cross-striations. The existence of normal fibroblasts and small connective tissue interstices between fibers indicates a healthy cardiac structure. These data show that Taif Rose extract alone does not induce any harmful structural effects on heart tissue and preserves normal histological integrity. These findings are consistent with previous reports demonstrating that naturally derived phytochemicals exert cardioprotective effects by attenuating oxidative stress and enhancing endogenous antioxidant defense mechanisms (Singaravel et al., 2025).

Conversely, Oral administration of lead acetate induced significant degenerative and necrotic alterations in the myocardium. Cardiac fibers exhibited disorganization and significant separation, accompanied by loss of striation and cytoplasmic vacuolation. The existence of pyknotic nuclei, uniform eosinophilic sarcoplasm, and engorged capillaries signifies significant cellular damage and vascular impairment. Masson’s trichrome staining revealed significant interstitial and perivascular fibrosis. These characteristics align with lead-induced myocardial toxicity documented in prior investigations, wherein lead exposure impaired cardiac fiber organization, may induce nuclear pyknosis, and resulted in interstitial edema due to heightened oxidative stress and calcium dysregulation (Xu et al., 2015). Lead ions can substitute for calcium in cardiac mitochondria and sarcolemma, disrupting ATP synthesis and contractile activity, resulting in the structural degeneration of cardiomyocytes (Minigaliyeva et al., 2024).

The deeply stained homogenous acidophilic (hypereosinophilic) sarcoplasm of many cardiomyocytes displayed in the current work agrees with Bahey (Bahey et al., 2019). Also, similar findings were reported in association with other cardiotoxic compounds, such as cyclophosphamide, gentamicin, and doxorubicin (Abdelatty et al., 2021). This hyper-eosinophilia may be ascribed to the enhanced affinity of eosin for damaged cytoplasmic proteins. The augmented protein breakdown may partially result from hydrolytic enzymes released by lysosomes in necrotic and apoptotic cells. The perinuclear sarcoplasmic vacuolation of cardiomyocytes seen in this study may be attributed to dilated T-tubules and sarcoplasmic reticulum, as suggested by Reis-Mendes et al. (Reis-Mendes et al., 2021). The present investigation effectively illustrated vascular problems, including congestion, dilation, red blood cell extravasation, bleeding, vacuolation, and thickening of blood channel walls. Prior research has corroborated the findings, linking lead exposure to an elevated risk of developing both coronary and peripheral artery diseases (Abdelatty et al., 2021).

The concurrent injection of Taif Rose extract with lead acetate significantly improved these histological changes. The myocardium exhibited a return to normal architecture and the preservation of nuclei. Only modest vascular congestion persisted, indicating partial yet considerable healing. The enhanced arrangement of myocardial fibers in this group indicates that Taif Rose extract has a stabilizing influence on heart cell membranes and intercellular connections, mitigating the oxidative and inflammatory damage consistent by lead exposure. The observed protection can be ascribed to the abundant bioactive compounds in the Taif Rose, notably flavonoids, phenolic acids, and essential oils, which exhibit significant antioxidant and anti-inflammatory effects (Yeddes et al., 2025). These chemicals are recognized for their ability to scavenge reactive oxygen species, augment natural antioxidant enzymes, including superoxide dismutase and glutathione peroxidase, and impede lipid peroxidation in cardiac tissue. Taif Rose extract likely mitigates oxidative stress, hence preventing mitochondrial malfunction and maintaining cellular integrity (Abdallah et al., 2023). Similar cardioprotective effects have been documented with additional plant-derived antioxidants, including green tea polyphenols, resveratrol, and curcumin, which mitigate structural cardiac damage in chemically induced cardiotoxicity (Alharbi et al., 2025).

This study demonstrated by Masson Trichrome findings, which demonstrated a substantial increase in the mean area of collagen fibers following chronic lead administration compared to the control group. This aligns with another study that attributed similar results to lipid peroxidation, which initiates an inflammatory response characterized by the excessive production of fibrogenic cytokines that promote fibrosis (Rajpoot et al., 2024). Conversely, co-treatment with Taif Rose markedly diminished the collagen area % to nearly normal levels, suggesting anti-fibrotic efficacy. The flavonoid components of Rosa damascena, including quercetin, kaempferol, and gallic acid, are recognized for their ability to suppress TGF-β–induced fibroblast activation and collagen synthesis, thus averting fibrotic remodelling (Zhang et al., 2022). Consequently, the reduction of fibrosis in the Taif Rose co-treated group may stem from the inhibition of oxidative stress-induced profibrotic signaling and the maintenance of cardiac extracellular matrix integrity.

Collectively, our data suggest that Taif Rose extract not only safeguards against myocardial hypertrophy but also aids in preserving overall physiological and structural cardiac integrity in the presence of heavy metal exposure. This indicates its possible therapeutic use as a natural cardioprotective drug to alleviate the harmful cardiovascular impacts of environmental toxins such as lead.

Cardiac troponin I (cTnI) and creatine kinase-MB (CK-MB) are established biomarkers of myocardial injury (Qi et al., 2025). The significant elevation of serum CK-MB and cTnI levels in lead-exposed rats indicates cardiomyocyte membrane damage and myocardial necrosis induced by oxidative stress. These findings are consistent with previous reports demonstrating that lead toxicity promotes sarcolemma disruption and leakage of intracellular cardiac enzymes into the circulation (Klinova et al., 2022). Increased cTnI levels further confirm myocardial injury under toxic stress conditions (Qi et al., 2025). In contrast, Taif Rose administration markedly restored CK-MB and cTnI levels, suggesting cardioprotective and membrane-stabilizing effects, likely may be mediated through attenuation of oxidative stress and preservation of cellular integrity.

Oxidative stress may induce by Pb exposure has been widely reported in experimental studies and is considered a key mechanism underlying lead toxicity (Vukelić et al., 2023). In the present study, lead acetate administration significantly increased malondialdehyde (MDA) levels while reducing antioxidant defenses (GSH and SOD), indicating enhanced lipid peroxidation and depletion of endogenous antioxidants. These findings are consistent with previous reports demonstrating that lead promotes reactive oxygen species (ROS) generation and impairs antioxidant enzyme systems in cardiac tissue (Fan et al., 2020). The resulting oxidative imbalance contributes to cellular and membrane damage, as reflected by elevated MDA levels observed in the Pb-exposed group (Javorac et al., 2022).

Mitochondria, often referred to as the powerhouses of the cell, are essential for maintaining cardiac and vascular function. They generate over 90% of myocardial adenosine triphosphate (ATP) through oxidative phosphorylation, regulate calcium homeostasis, and modulate ROS production. Dysregulation of these processes plays a significant role in the development and progression of endothelial dysfunction, inflammatory cascades, and cardiomyocyte injury, which are hallmarks of CVDs. Pb disrupts mitochondrial function and activates pro-inflammatory signaling pathways (Yang, 2025).

The co-administration of Taif Rose extract markedly diminished MDA and superoxide levels while reinstating reduced glutathione (GSH) content and antioxidant enzyme activity to near-normal levels. This suggests its antioxidant and free radical scavenging potential, which significantly mitigate Pb-induced oxidative damage. Similar antioxidative effects of *Rosa damascena* have been reported by Boskabady (Boskabady et al., 2011), Trendafilova (Trendafilova et al., 2023), and Chandana (Chandana et al., 2024), who attributed them to its high phenolic and flavonoid content, including quercetin and kaempferol derivatives.

These findings suggest that oxidative stress is a primary associated with of lead-induced cardiotoxicity and that Taif Rose extract may exert protective effects by enhancing antioxidant defences, maintaining sulfhydryl status, and restoring redox balance. These effects may contribute to the observed reduction in cardiac hypertrophy and fibrosis in treated animals. This study has some limitations. Only male rats were included to minimize hormonal variability associated with the estrous cycle; therefore, potential sex-related differences were not evaluated. In addition, only a single dose of lead acetate and Rosa damascena extract was investigated, precluding assessment of dose–response relationships or the optimal therapeutic dose. Future studies incorporating molecular approaches, such as gene and protein expression analyses, are warranted to validate these mechanisms.

## 6 Conclusion

This study demonstrates that prolonged exposure to lead acetate induces significant myocardial injury, as evidenced by increased oxidative stress, degeneration of cardiac muscle fibers, and collagen accumulation. Co-treatment with Taif Rose leaf and stem extract offered significant protection against these harmful effects, as seen by the normalization of biochemical indicators, maintenance of histological integrity, and reduction of fibrosis. The cardioprotective action of Taif Rose is due to its abundant polyphenols and flavonoids, which neutralize free radicals, bolster endogenous antioxidant defences, and regulate inflammatory and fibrotic responses.

Consequently, the Taif Rose is a promising candidate for the advancement of cardioprotective medicines, particularly among populations susceptible to environmental heavy metal exposure.

## Data Availability

The original contributions presented in the study are included in the article/supplementary material, further inquiries can be directed to the corresponding author.

## References

[B1] Abdallah H. M. Koshak A. E. Farag M. A. El Sayed N. S. Badr-Eldin S. M. Ahmed O. A. A. (2023). Taif rose oil ameliorates UVB-Induced oxidative damage and skin photoaging in rats via modulation of MAPK and MMP signaling pathways. ACS Omega 8 (37), 33943–33954. 10.1021/acsomega.3c04756 37744837 PMC10515598

[B2] Abdelatty A. Ahmed M. S. Abdel-Kareem M. A. Dmerdash M. Mady R. Saad A. S. (2021). Acute and delayed doxorubicin-induced myocardiotoxicity associated with elevation of cardiac biomarkers, depletion of cellular antioxidant enzymes, and several histopathological and ultrastructural changes. Life 11 (9), 880. 10.3390/life11090880 34575029 PMC8467687

[B3] Abdulhai F. Motairek I. Mirzai S. Bazarbachi B. Chamseddine F. Alamer M. (2024). Quantifying lead-attributable cardiovascular disease burden in the United States. Curr. Probl. Cardiol. 49 (6), 102565. 10.1016/j.cpcardiol.2024.102565 38599559

[B4] Al-Juboori B. Hamdan F. Al-Salihi A. (2016). Paternal exposure to low-dose lead acetate: effect on implantation rate,pregnancy outcome, and sex ratio in mice. Turk. J. Med. Sci. Apr 46 (3), 936–941. 10.3906/sag-1412-62 27513276

[B5] Alharbi H. O. A. Alshebremi M. Babiker A. Y. Rahmani A. H. (2025). The role of quercetin, a flavonoid in the management of pathogenesis through regulation of oxidative stress, inflammation, and biological activities. Biomolecules 15 (1), 151. 10.3390/biom15010151 39858545 PMC11763763

[B6] Bahey N. G. Abd Elaziz H. O. Gadalla K. K. E. (2019). Potential toxic effect of bisphenol A on the cardiac muscle of adult rat and the possible protective effect of Omega-3: a histological and immunohistochemical study. J. Microsc. Ultrastruct. 7 (1), 1–8. 10.4103/jmau.jmau_53_18 31008050 PMC6442328

[B7] Boskabady M. H. Shafei M. N. Saberi Z. Amini S. (2011). “Pharmacological effects of rosa damascena,”Iran. J. Basic Med. Sci. 14. 295–307. 10.22038/IJBMS.2011.5018 23493250 PMC3586833

[B8] Chandana J. V. S. Kumar G. A. Vaishnavi B. Adarsh A. Rao T. R. (2024). A review on antioxidant activity of Rosa damascena. Int. J. Res. Rev. 11 (3), 156–162. 10.52403/ijrr.20240321

[B9] Chen Z. Huo X. Chen G. Luo X. Xu X. (2021). Lead (Pb) exposure and heart failure risk. Environ. Sci. Pollut. Res. 28 (23), 28833–28847. 10.1007/s11356-021-13725-9 33840028

[B10] Desai S. Wilson J. Ji C. Sautner J. Prussia A. J. Demchuk E. (2024). The role of simulation science in public health at the agency for toxic substances and disease registry: an overview and analysis of the last decade. Toxics 12 (11), 811. 10.3390/toxics12110811 39590991 PMC11598116

[B11] Etten V. (2002). Recommended methods of anesthesia, analgesia, and euthanasia for laboratory animal species. Inst. Anim. Stud. 460 (718), 839–7100.

[B12] Fan Y. Zhao X. Yu J. Xie J. Li C. Liu D. (2020). Lead-induced oxidative damage in rats/mice: a meta-analysis. J. Trace Elem. Med. Biol. 58, 126443. 10.1016/j.jtemb.2019.126443 31841831

[B13] Ferreira de Mattos G. Costa C. Savio F. Alonso M. Nicolson G. L. (2017). Lead poisoning: acute exposure of the heart to lead ions promotes changes in cardiac function and Cav1.2 ion channels. Biophys. Rev. 9 (5), 807–825. 10.1007/s12551-017-0303-5 28836190 PMC5662044

[B14] Gioda A. Beringui K. Justo E. P. S. Ventura L. M. B. Massone C. G. Costa S. S. L. (2022). A review on atmospheric analysis focusing on public health, environmental legislation and chemical characterization. Crit. Rev. Anal. Chem. 52 (8), 1772–1794. 10.1080/10408347.2021.1919985 34092145

[B15] Gosselin R. E. Smith R. P. Hodge H. C. Braddock J. E. (1984). Clinical Toxicology of Commercial Products, 1085. Baltimore: Williams and Wilkins.

[B16] Hakimi F. Eidi A. Ehsani E. Ayyari M. Shahpasand K. (2025). Neuroprotective effects of Rosa damascena essential oil against ethanol-induced toxicity in neural progenitor cells. Jundishapur J. Nat. Pharm. Prod. 20 (3), e160675. 10.5812/jjnpp-160675

[B17] Hamza R. Z. Al-Yasi H. M. Ali E. F. Fawzy M. A. Abdelkader T. G. Galal T. M. (2022). Chemical characterization of taif rose (Rosa damascena mill Var. trigentipetala) waste methanolic extract and its hepatoprotective and antioxidant effects against cadmium chloride (CdCl2)-induced hepatotoxicity and potential anticancer activities against. Crystals 12 (4), 460. 10.3390/cryst12040460

[B18] Harshitha P. Bose K. Dsouza H. S. (2024). Influence of lead-induced toxicity on the inflammatory cytokines. Toxicology 503, 153771. 10.1016/j.tox.2024.153771 38452865

[B19] He A. Putra N. R. (2025). Rose essential oils: current trends, mapping of extraction techniques, chemical analysis, therapeutic applications, and by‐product valorization. Can. J. Chem. Eng. 103 (7), 3332–3357. 10.1002/cjce.25554

[B20] Hedayati N. Yaghoobi A. Salami M. Gholinezhad Y. Aghadavood F. Eshraghi R. (2023). Impact of polyphenols on heart failure and cardiac hypertrophy: clinical effects and molecular mechanisms. Front. Cardiovasc Med. 10, 1174816. 10.3389/fcvm.2023.1174816 37293283 PMC10244790

[B21] Janicka P. Stygar D. Chełmecka E. Kuropka P. Mi azek A. Studzi nska A. (2024). Oxidative stress markers and histopathological changes in selected organs of mice infected with murine norovirus 1 (MNV-1). Int. J. Mol. Sci. 25, 3614. 10.3390/ijms25073614 38612426 PMC11011583

[B22] Javorac D. Tatović S. Anđelković M. Repić A. Baralić K. Djordjevic A. B. (2022). Low-lead doses induce oxidative damage in cardiac tissue: subacute toxicity study in wistar rats and benchmark dose modelling. Food Chem. Toxicol. 161, 112825. 10.1016/j.fct.2022.112825 35045334

[B23] Jomova K. Alomar S. Y. Nepovimova E. Kuca K. Valko M. (2025). Heavy metals: toxicity and human health effects. Arch. Toxicol. Jan. 99 (1), 153–209. 10.1007/s00204-024-03903-2 39567405 PMC11742009

[B24] Klinova S. V. Minigalieva I. A. Protsenko Y. L. Sutunkova M. P. Gurvich V. B. Ryabova J. V. (2022). Changes in the cardiotoxic effects of lead intoxication in rats induced by muscular exercise. Int. J. Mol. Sci. Apr 23 (8), 4417. 10.3390/ijms23084417 35457235 PMC9029617

[B25] Kumar V., S, H. Huligowda L. K. D. Umesh M. Chakraborty P. Thazeem B. (2025). Environmental pollutants as emerging concerns for cardiac diseases: a review on their impacts on cardiac health. Biomedicines 13, 241. 10.3390/biomedicines13010241 39857824 PMC11759859

[B26] Lalovski I. Krasteva M. Yordanov E. Hristov E. Dimitrov M. Parvova I. (2025). Safety of active substances derived from Rosa damascena and their potential biological activity in humans: a systematic review. Pharmacia 72, 1–10. 10.3897/pharmacia.72.e165898

[B27] Lamas G. A. Bhatnagar A. Jones M. R. Mann K. K. Nasir K. Tellez Plaza M. (2023). Contaminant metals as cardiovascular risk factors: a scientific statement from the American heart association. J. Am. Heart Assoc. 12 (13), e029852. 10.1161/JAHA.123.029852 37306302 PMC10356104

[B28] Lowry O. H. Rosebrough A. Far L. Randall R. J. (1951). Protein measurement with the folin phenol reagent. J. Biol. Chem. 193 (1), 265–275. 10.1016/0021-9258(51)90565-2 14907713

[B29] Minigaliyeva I. A. Klinova S. V. Sutunkova M. P. Ryabova Y. V. Valamina I. E. Shelomentsev I. G. (2024). On the mechanisms of the cardiotoxic effect of lead oxide nanoparticles. Cardiovasc Toxicol. 24 (1), 49–61. 10.1007/s12012-023-09814-5 38108959 PMC10838250

[B30] Neha V. Parithathvi A. Dsouza H. S. (2025). Ameliorative role of bioactive compounds against lead-induced neurotoxicity. Neuroscience 568, 46–56. 10.1016/j.neuroscience.2025.01.018 39805419

[B31] Protsenko Y. L. Katsnelson B. A. Klinova S. V. Lookin O. N. Balakin A. A. Nikitina L. V. (2018). Effects of subchronic lead intoxication of rats on the myocardium contractility. Food Chem. Toxicol. 120, 378–389. 10.1016/j.fct.2018.07.034 30036551

[B32] Protsenko Y. L. Klinova S. V. Gerzen O. P. Privalova L. I. Minigalieva I. A. Balakin A. A. (2020). Changes in rat myocardium contractility under subchronic intoxication with lead and cadmium salts administered alone or in combination. Toxicol. Reports 7, 433–442. 10.1016/j.toxrep.2020.03.001 32181144 PMC7063142

[B33] Qi Y. Cai M. Kang Y. Liu J. (2025). Progress in biomarkers and diagnostic approaches for myocardial infarction. Clin. Chim. Acta 579, 120629. 10.1016/j.cca.2025.120629 41005554

[B34] Radhiga T. Rajamanickam C. Senthil S. Pugalendi K. V. (2012). Effect of ursolic acid on cardiac marker enzymes, lipid profile and macroscopic enzyme mapping assay in isoproterenol-induced myocardial ischemic rats. Food Chem. Toxicol. 50 (11), 3971–3977. 10.1016/j.fct.2012.07.067 22898613

[B35] Rajpoot A. Aggarwal T. Sharma V. (2024). Unraveling the enigma of cardiac damage caused by lead: understanding the intricate relationship between oxidative stress and other multifactorial mechanisms. Toxicology 509, 153984. 10.1016/j.tox.2024.153984 39481524

[B36] Reis-Mendes A. Padrão A. I. Duarte J. A. Gonçalves-Monteiro S. Duarte-Araújo M. Remião F. (2021). Role of inflammation and redox status on doxorubicin-induced cardiotoxicity in infant and adult CD-1 male mice. Biomolecules 11 (11), 1725. 10.3390/biom11111725 34827723 PMC8615472

[B37] Riaz M. Khalid R. Afzal M. Anjum F. Fatima H. Zia S. (2023). Phytobioactive compounds as therapeutic agents for human diseases: a review. Food Sci. Nutr. 11 (6), 2500–2529. 10.1002/fsn3.3308 37324906 PMC10261751

[B38] Singaravel V. Murugan P. Moovendhan M. (2025). Brazilin-enriched Caesalpinia sappan heartwood extract mitigates Doxorubicin-induced cardiotoxicity. Pharmacol. Res. - Mod. Chin. Med. 16, 100667–100674. 10.1016/j.prmcm.2025.100667

[B39] Suvarna K. S. Layton C. Bancroft J. D. (2018). Bancroft’s Theory and Practice of Histological Techniques. 7th ed. Elsevier Health Sciences, 215–236.

[B40] Swaringen B. F. Gawlik E. Kamenov G. D. McTigue N. E. Cornwell D. A. Bonzongo J.-C. J. (2022). “Children’s exposure to environmental lead: a review of potential sources, blood levels, and methods used to reduce exposure,”Environ. Res., 204. 10.1016/j.envres.2021.112025 34508773

[B41] Tietz F. (1969). Enzymic method for quantitative determination of nanogram amounts of total and oxidized glutathione: applications to mammalian blood and other tissues. Anal. Biochem. 27 (3), 502–522. 10.1016/0003-2697(69)90064-5 4388022

[B42] Trendafilova A. Staleva P. Petkova Z. Ivanova V. Evstatieva Y. Nikolova D. (2023). “Phytochemical profile, antioxidant potential, antimicrobial activity, and cytotoxicity of dry extract from Rosa damascena mill,”Molecules, 28. 7666. 10.3390/molecules28227666 38005389 PMC10674922

[B43] Vukelić D. Djordjevic A. B. Anđelković M. Antonijević Miljaković E. Baralić K. Živančević K. (2023). Subacute exposure to low Pb doses promotes oxidative stress in the kidneys and copper disturbances in the liver of Male rats. Toxics 11 (3), 256. 10.3390/toxics11030256 36977021 PMC10056143

[B44] World Health Organization W (2024). Exposure to lead: a major public health concern. Preventing Disease Through Healthy Environments. Geneva, Switzerland: World Health Organization. Available online at: https://books.google.com.sa/books?hl=ar&lr=&id=z6MOEQAAQBAJ&oi=fnd&pg=PA1 (Accessed August 2, 2026).

[B45] Xu L.-H. Mu F.-F. Zhao J.-H. He Q. Cao C.-L. Yang H. (2015). Lead induces apoptosis and histone hyperacetylation in rat cardiovascular tissues. PLoS One 10 (6), e0129091. 10.1371/journal.pone.0129091 26075388 PMC4468051

[B46] Yang H.-M. (2025). Mitochondrial dysfunction in cardiovascular diseases. Int. J. Mol. Sci. 26 (5), 1917. 10.3390/ijms26051917 40076543 PMC11900462

[B47] Yeddes W. Reguez S. Betaieb Rebey I. Wannes W. A. Majdi H. Dakhlaoui S. (2025). Valorisation of hydrodistillation by-products from damask rose (Rosa damascena): extraction, characterization, and bioactivity of phenolic compounds with biological properties. Int. J. Environ. Health Res. 35, 1–9. 10.1080/09603123.2025.2491634 40232048

[B48] Yilgor A. Demir C. (2024). Determination of oxidative stress level and some antioxidant activities in refractory epilepsy patients. Sci. Rep. 14 (1), 6688. 10.1038/s41598-024-57224-6 38509121 PMC10954705

[B49] Zhang H. Yang L. Han Q. Xu W. (2022). Antifibrotic effects of quercetin on TGF-β1-induced vocal fold fibroblasts. Am. J. Transl. Res. 14 (12), 8552–8561. 36628236 PMC9827314

